# Supplementary material for: The Role of Plasminogen Activator Inhibitor 1 in Predicting Sepsis-Associated Liver Dysfunction: An Observational Study
Source: Int J Environ Res Public Health. 2023 Mar 9;20(6):4846. doi: 10.3390/ijerph20064846 (PMC10049524; doi:10.3390/ijerph20064846)
Supplement: Supplementary file 1 [file ijerph-20-04846-s001.zip › ijerph-2205212-Supplementary.pdf]

Supplementary Materials

Correlation between PAI-1 and selected variables

Table 1supp shows the correlation between PAI-1 levels on day 1. of observation and bilirubin levels, SOFA score and lactate, procalcitonin, AST and ALP levels in the following days of observation in the whole study group.

Table S1. Correlation between PAI-1 levels on day 1. of observation, selected variables in the following days of observation in the whole study group.

Day of observation	Marker	Spearman correlation			Marker	Spearman correlation			Marker	Spearman correlation		
		N	R	P		N	R	P		N	R	P
1	**Bilirubin**	76	0.26	0.02	**SOFA**	79	0.26	0.020	**Lactate**	79	0.63	<0.001
3	**Bilirubin**	69	0.42	<0.001	**SOFA**	70	0.37	0.0021	**Lactate**	70	0.50	<0.001
5	**Bilirubin**	53	0.46	0.001	**SOFA**	55	0.39	0.003	**Lactate**	55	0.43	0.001
7	**Bilirubin**	48	0.43	0.003	**SOFA**	51	0.29	0.037	**Lactate**	51	0.35	0.012
14	**Bilirubin**	19	0.29	0.22	**SOFA**	21	0.13	0.57	**Lactate**	21	0.01	0.98
1	**Procalcitonin**	78	0.21	0.07	AST	74	0.42	<0.001	**ALP**	56	0.16	0.252
3	**Procalcitonin**	70	0.46	<0.001	AST	62	0.55	<0.001	**ALP**	17	0.08	0.758
5	**Procalcitonin**	54	0.35	0.010	AST	45	0.57	<0.001	**ALP**	13	0.12	0.707
7	**Procalcitonin**	50	0.30	0.035	AST	45	0.60	<0.001	**ALP**	13	0.18	0.553
14	**Procalcitonin**	21	-0.16	0.48	AST	17	0.64	0.006	**ALP**	5	0.90	0.037
